# Clinical outcomes of tibial tuberosity transposition with a controlled translation device for the treatment of patellar luxation in dogs: fifteen surgeries on 14 cases (2021‐2022)

**DOI:** 10.1111/jsap.13890

**Published:** 2025-06-12

**Authors:** R. C. de Souza Faustino, E. H. P. Curuci, L. V. Costa, B. W. Minto, L. G. G. G. Dias

**Affiliations:** ^1^ Department of Clinical and Veterinary Surgery, School of Agricultural and Veterinary Sciences “Júlio de Mesquita Filho” São Paulo State University – UNESP Jaboticabal Brazil; ^2^ OrtopediaVet Veterinary Clinic, Dr. Eloy Curuci and Team São Paulo Brazil

## Abstract

**Objectives:**

The aim of this study was to evaluate the efficacy and outcomes of the tibial tuberosity transposition technique modified using a slow and controlled translation device (mTTT), in dogs with patellar luxation. The technique seeks to realign the quadriceps extensor mechanism using a dedicated device for tibial tuberosity transposition.

**Materials and Methods:**

Fourteen dogs with grade II medial patellar luxation were included in the study, and 15 stifles were treated using this technique. A partial osteotomy of the tibial tuberosity was performed, followed by its transposition using a dedicated device. Finally, the transposition was stabilized with a pin spacer inserted between the tibial tubercle and the medial cortex of the tibia. Realignment of the quadriceps extensor mechanism was visually evaluated at the end of the surgical procedure and in post‐operative radiographic images. The animals were evaluated for the degree of post‐operative lameness, quality and time of consolidation of the osteotomy.

**Results:**

No animal experienced patellar reluxation after the procedure. Two complications (13.33%) were recorded: one minor (6.66%), represented by a fracture of the osteotomized fragment during tuberosity translation, and one major (6.66%), involving implant migration observed in the post‐operative period. One patient (6.66%) showed grade I lameness at 30 days, but the others did not present lameness (grade 0). At 60 days, no lameness was observed in any animal. Bone consolidation was considered excellent in 12 cases (80%), good in one case (6.66%) and reasonable in two cases (13.33%) at 30 days after the surgery and excellent in all cases at 60 days.

**Clinical Significance:**

The results confirm that the mTTT technique is an effective alternative with excellent results and low complication rates in treating dogs with grade II medial patellar luxation.

## INTRODUCTION

Patellar luxation (PL) is one of the main disorders of the canine stifle and one of the most common causes of lameness in the pelvic limbs (Bound et al., 2009; Roush, 1993). PL is characterized by the pathological displacement of the patella in relation to the trochlear sulcus (L’Eplattenier & Montavon, 2002; Roush, 1993). The condition has complex, multifactorial causes and is triggered by a set of musculoskeletal changes that occur during the patient’s development, affecting the structures that constitute the quadriceps extensor mechanism (QEM). Typically, it is diagnosed in young dogs of small or miniature breeds; however, clinical signs are usually more evident after musculoskeletal maturity (Alam et al., 2007; Bound et al., 2009; O’Neill et al., 2016). Medial patellar luxation (MPL) is more frequent than lateral PL and represents approximately 80% of diagnoses (Bosio et al., 2017; Di Dona et al., 2018; Perry & Déjardin, 2021).

Surgical treatment is especially recommended for patients with pain, recurrent or permanent lameness and postural alterations (Kowaleski et al., 2017; Piermattei et al., 2006). The goal of surgery is to re‐establish the alignment of the QEM, giving stability to the patella over the femoral trochlea (Cashmore et al., 2014; Kowaleski et al., 2017). Surgical techniques are aimed at correcting bone deformities that are beyond normal limits and are based on the degree of luxation, the extent and type of bone malformation and soft tissue changes (Arthurs & Langley‐Hobbs, 2006; Kowaleski et al., 2017). Among the most commonly used techniques are tibial tuberosity transposition (TTT), femoral trochleoplasty and soft tissue procedures such as medial capsule release and lateral capsule imbrication (Arthurs & Langley‐Hobbs, 2006; Piermattei et al., 2006; Willauer & Vasseur, 1987).

TTT is a bone alignment correction technique initially described by Singleton in 1957 (Singleton, 1969). It realigns the QEM based on the translation of the tibial tuberosity (TT) to correct tibial torsion. It is the main technique used to correct angular deviations involving the TT and can be considered one of the most important steps in the surgical correction of PL (Alam et al., 2007; Linney et al., 2011). Over the years, several methods involving different techniques of osteotomy and fixation of the TT have been described to achieve acceptable results (Cashmore et al., 2014; Kowaleski et al., 2017; Palmer, 2009; Piermattei et al., 2006; Pugliese et al., 2015; Roush, 1993; Schulz, 2007; Stanke et al., 2014). However, complications in the surgical treatment of PL have been described (Arthurs & Langley‐Hobbs, 2006; Cashmore et al., 2014; Kowaleski et al., 2017; Linney et al., 2011; Petazzoni, 2010; Schulz, 2007) and can sometimes exceed acceptable limits. Severe complications requiring surgical re‐intervention, such as patellar reluxation, delayed union, implant failure/migration, osteotomy fixation failure, fracture and/or avulsion of the tibial crest, occur in 13% to 25% of the cases (Arthurs & Langley‐Hobbs, 2006; Cashmore et al., 2014; Kowaleski et al., 2017; Linney et al., 2011; Stanke et al., 2014). Minor complications, such as infection or dehiscence of the suture, seroma formation, inflammation of the patellar ligament, hyperextension of the tibiotarsal joint and lameness resulting from osteoarthrosis or irritation caused by TTT surgical implants, occur in an additional 5% of the cases (Arthurs & Langley‐Hobbs, 2006; Harasen, 2006).

Petazzoni (2015) described the “Tibial Tuberosity Transposition Tool” (TTTT®) technique with the aim of reducing trans‐ and post‐surgical complications reported with TTT. This treatment is recommended for patients with grade I or II PL and is performed using a special device that enables slow and controlled transposition of the TT, which is stabilized at the end by a Steinmann pin that does not transfix the TT but is positioned adjacent to it. The procedure enhances tuberosity resistance and bone consolidation, while also reducing the need for implants. Despite its routine use by veterinary surgeons globally, studies clinically validating the technique are scarce. Therefore, this case series aims to describe the clinical and radiographic results of 14 dogs with grade II MPL treated using the TTT technique modified with a slow and controlled translation device (mTTT). It is postulated that the use of mTTTmay provide more satisfactory outcomes, such as early consolidation and recovery, along with lower complication rates, including delayed union, implant failure/migration, osteotomy fixation failure, fracture and/or avulsion of the tibial crest and skin irritation or seroma, when compared to traditional TTT techniques.

## MATERIALS AND METHODS

Medical records of procedures performed between 2021 and 2022 at a centre specialized in veterinary orthopaedics were reviewed. Dogs with grade I or II MPL and estimated external tibial torsion of <20° treated with the TTT technique modified using a slow and controlled translation device (mTTT) were included in the study.

### Case selection

The inclusion criteria were dogs with grade I or II PL evaluated in both lateral recumbency and in the quadrupedal position, regardless of breed, sex or weight. The animals underwent a comprehensive clinical and orthopaedic evaluation. Dogs with a history of traumatic injuries or with orthopaedic or systemic diseases that could compromise the study results, such as fractures, joint luxations, osteoarthritis, infectious diseases and metabolic disorders, were excluded. Additionally, dogs with an anatomical lateral distal femoral angle >96° or with external/internal tibial torsion exceeding 20° were excluded from the study.

### Treatment

The surgical planning was performed based on a digital radiographic study of the affected pelvic limb using a 2.54‐cm spherical magnifier. The patient was placed under sedation or general anaesthesia. Femoral deviations were assessed using mediolateral and craniocaudal projections. Proximal tibial torsion was evaluated from mediolateral and craniocaudal radiographic images of the tibia, including the femoro‐tibial‐patellar and tibio‐tarsal joints. The external/internal tibial torsion was estimated by comparing the craniocaudal projections with reference images for evaluation of the internal/external tibial torsion created by Petazzoni and Jaeger (2008).

**FIG 1:**
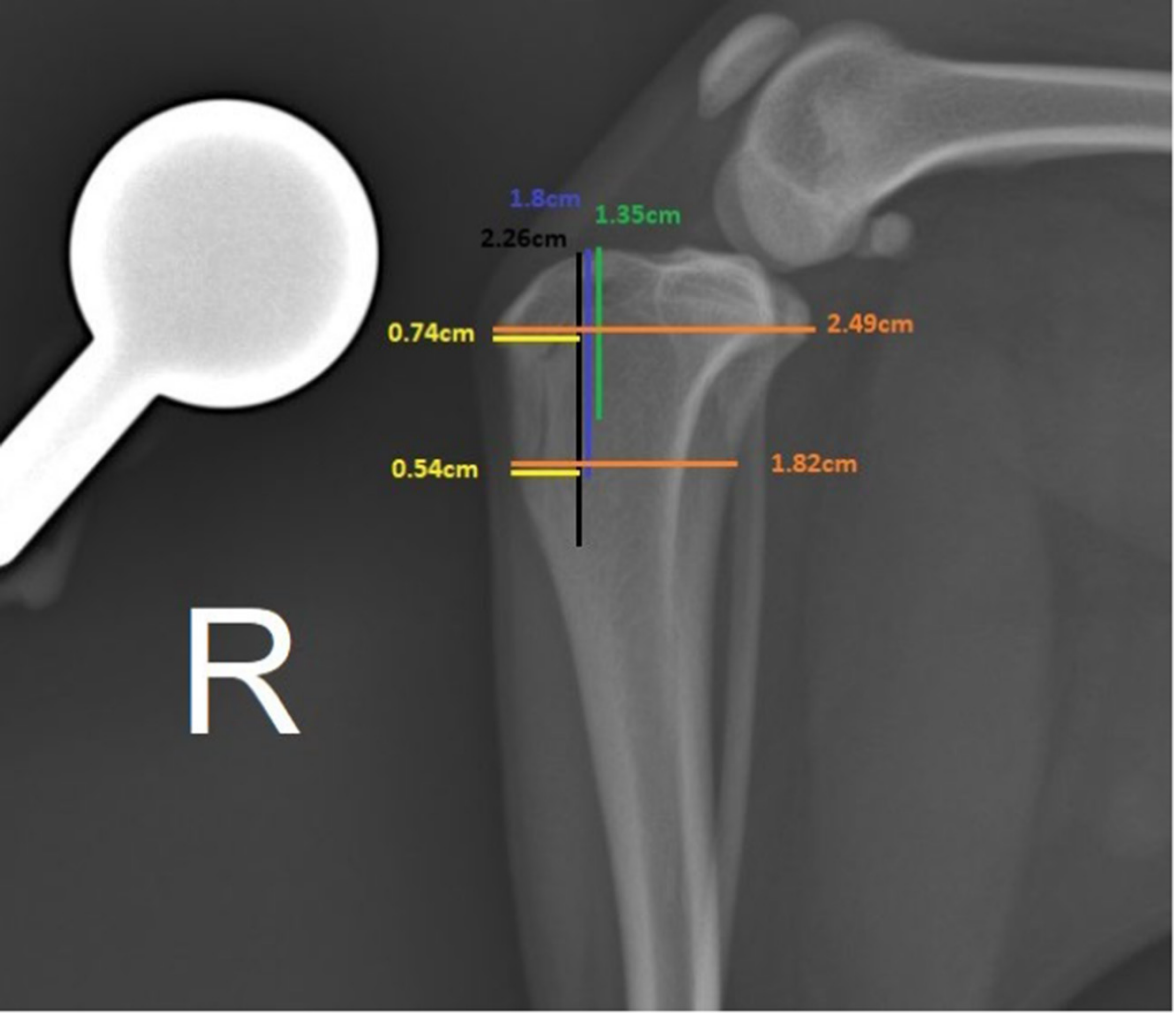
Planning of tibial tuberosity osteotomy in the sagittal plane. The blue and green lines correspond to the tibial tuberosity osteotomy at its medial and lateral cortex, respectively. Black line, 100% of the tibial tuberosity length; blue line, 80% of the tibial tuberosity length; green line, 60% of the tibial tuberosity length; orange lines, width of proximal tibia; yellow lines, 30% of width of proximal tibia.

As described by Petazzoni (2015), using the mediolateral projection of the tibia, the TT was outlined and measured for planning a partial osteotomy (Fig 1). Using a fine oscillating saw, oriented from proximal to distal and from medial to lateral, the osteotomy was initiated proximally at the level of Gerdy’s tubercle and continued distally, provided the planned 30% of the width of the tibia was maintained. The distal region connecting the TT to the rest of the diaphysis was left intact. In lateral transposition, the osteotomy in the medial cortex extended for 80% of the total length of the TT, oriented from proximal to distal. The lateral cortex was osteotomized for 60% of the total length of the TT. In the sagittal plane, the osteotomy aimed to achieve a TT width of 30% of the proximal tibia.

**FIG 2:**
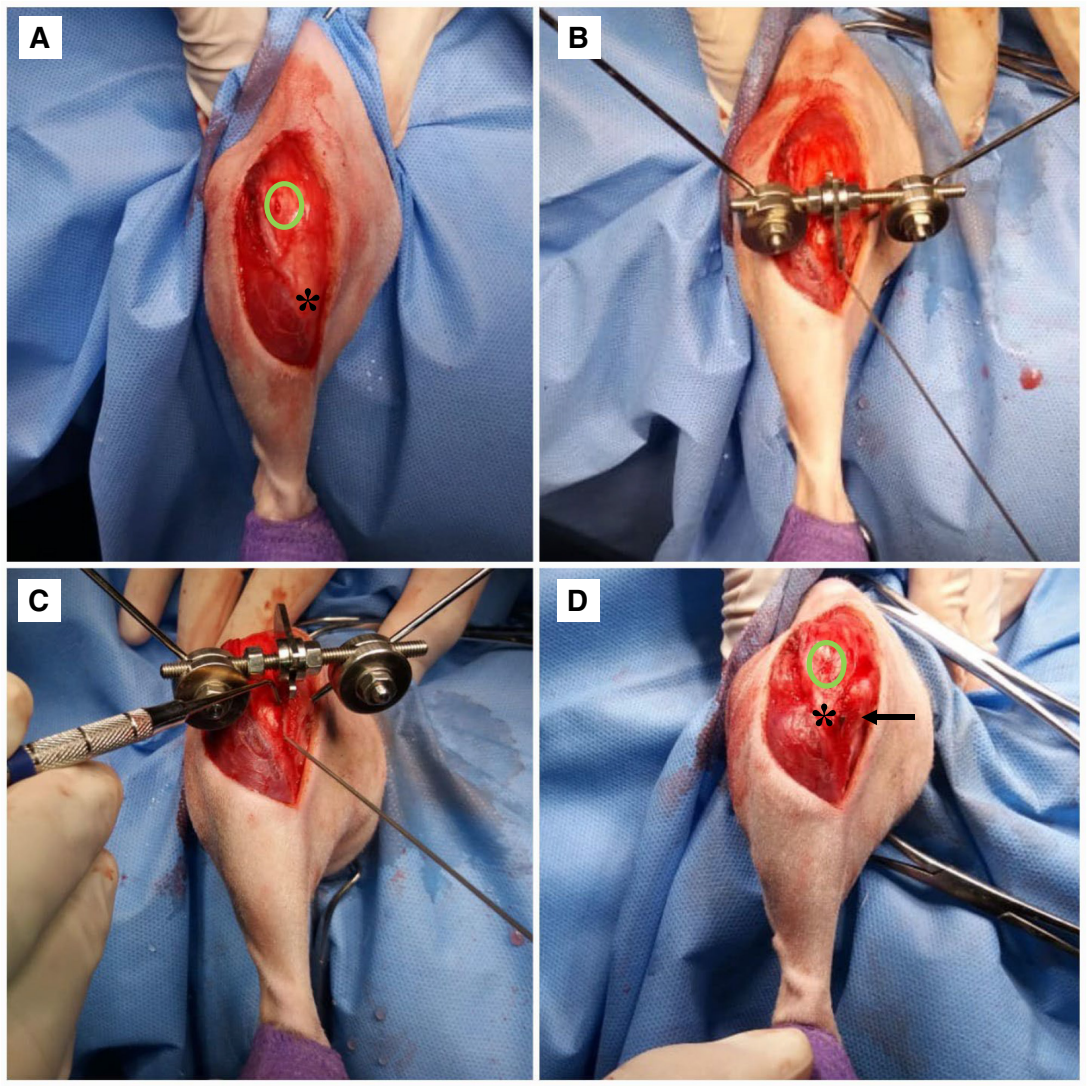
(A) Transoperative image, surgical access with a craniomedial skin incision (green circle patella, black asterisk tibial tuberosity); (B) transoperative image demonstrating the application and translation performed by the device, composed of a horizontal threaded bar with a “central sliding flap” and an adjacent nut, connected to two pins through spherical joints; (C) translation was periodically monitored with the aid of a specimeter during the intraoperative period to ensure controlled adjustment; (D) final result of the technique, with a subcortical Steinmann pin was planced adjacent to the tibial tuberosity in a cranial‐to‐caudal orientation, with an inclination similar to the angle of the tibial plateau in the sagittal plane, touching but not penetrating the trans cortex (black arrow).

The surgical procedures were performed by the same surgeon, who has 20 years of experience in veterinary surgery and orthopaedics. A craniomedial skin incision was made, starting at the level of the patella and extending to the most distal portion of the TT for surgical access (Fig 2A). If necessary, a wedge trochleoplasty was performed to deepen the trochlear sulcus.

After the wedge trochleoplasty, a partial osteotomy of the TT in the frontal plane was performed, oriented from proximal to distal and from medial to lateral, creating a bony pedicle for subsequent transposition.

**FIG 3:**
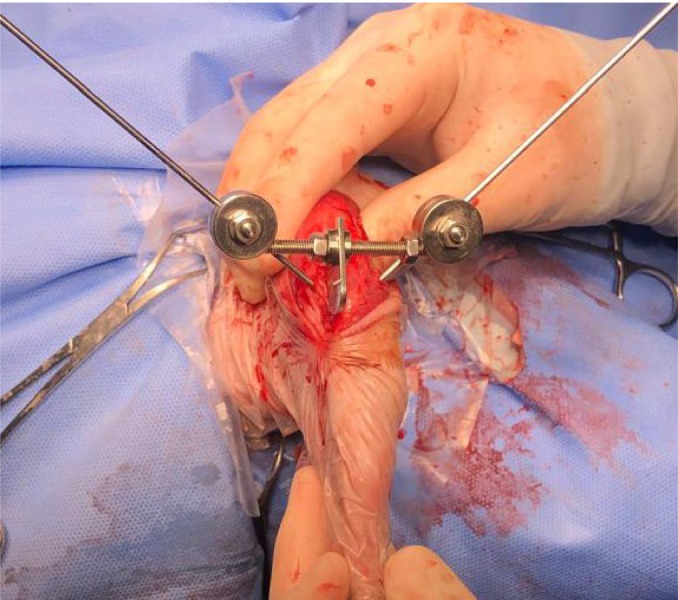
TTTT® device assembled in the postoperative period. Composed of a horizontal threaded bar; central sliding flap; two nuts; two spherical joints.

After osteotomy of the tibial crest, the TTTT® device (Intrauma SPA Turin, Italy by Petazzoni) was temporarily applied according to the technique described (Fig 3). After adjusting the device, the nut must be turned in order to push the central grooved piece and achieve the desired transposition (Fig 2B).

Transposition of the tibial crest was performed gradually until the alignment of the QEM was visually achieved. The desired transposition was accomplished by rotating the central tab with the device’s nut at a rate of one turn per minute (0.7 mm/minute), ideally a quarter turn every 15 seconds, to ideally minimize the risk of fracturing the displaced TT. Upon completion of the transposition, the stability and patellar alignment with the femoral trochlea and TT were visually assessed by performing stifle flexion/extension and external/internal rotation of the tibia relative to the femur (Fig 2C). When necessary, adjustments to the tuberosity position were made by turning the nut in the required direction to achieve realignment.

Before removing the transposition device, *t* a subcortical pin was then inserted at the same level of the patellar ligament insertion, between the inner edge of the cortex of the osteotomized tibia and the outer edge of the cortex of the TT. The Steinmann pin was placed in a cranial‐to‐caudal orientation, with an inclination similar to the angle of the tibial plateau in the sagittal plane, and advanced until it reached the caudal cortex without perforating it, to prevent potential caudal migration of the pin postoperatively. The pin is maintained in position by the pressure exerted by the tibial cortices during bone healing. The Steinmann pin was then sectioned caudally to the cranial surface of the TT (Fig 2D).

### Radiographic follow‐up

**FIG 4:**
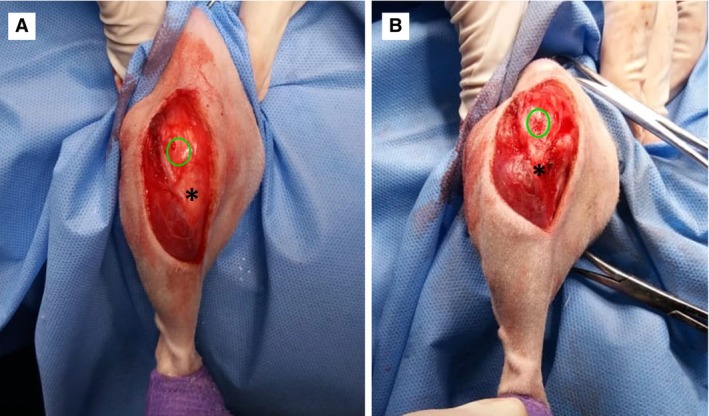
(A) Transoperative view before transposition, misalignment of the quadriceps extensor mechanism (green circle patella, black asterisk tibial tuberosity); (B) transoperative view after transposition, realignment of the quadriceps extensor mechanism (green circle patella, black asterisk tibial tuberosity).

Alignment was then re‐assessed immediately after the procedure (Fig 4). Radiographs were obtained in the craniocaudal and mediolateral projections, including views of the stifle and tarsal joints. Subsequent radiographic evaluations were performed every 30 days postoperatively until bone consolidation was evident, or more frequently at the surgeon’s discretion. The images were used to assess the proper execution of the technique and the progression of bone consolidation according to the classification criteria proposed by the International Society for Limb Salvage, where scores were defined as follows: 1¼ poor union, with <25% cure (no evidence of callus), 2¼ tight union with 25% to 50% cure, 3¼ good bond with cure more than 50% to 75% and 4¼ excellent bond with more than 75% healing (Table 1). Additionally, the translation of the TT achieved through the transposition technique was measured in the same radiographic projections.

**Table 1:** Classification of radiographic bone scarring

Score	Radiographic evaluation
Bad union	<25% scarring (no evidence of callus)
Reasonable union	>25% and <50% healing
Good union	>50% and <75% healing
Excellent union	>75% healing

Glaser and Langlais (1991)

### Post‐surgical, clinical and orthopaedic follow‐up

Between 10 and 15 days post‐surgery, patients underwent their first evaluation. Subsequent evaluations were performed every 30 days until complete bone consolidation was evident. During the orthopaedic examination, the stifle joint was assessed for pain during flexion and hyperextension, pain on deep palpation, crepitation and patellar reluxation. Patellar stability and alignment were evaluated by performing stifle flexion/extension movements and external/internal rotation of the tibia relative to the femur, with patients in both lateral decubitus and quadrupedal positions. The realignment of the QEM was visually assessed by observing the linear alignment of the patella, distal femur and TT in the frontal view of the pelvic limb during flexion and extension movements. Patient ambulation was consistently evaluated by the same observer during walking and trotting. The degree of lameness and levels of discomfort or pain were scored using a scale adapted from McCarthy et al. (2007) (Table 2). Other changes or complications were recorded and classified as minor or major, as described by Cook et al. (2010).

**Table 2:** Lameness assessment scale in dogs

Scoring system for evaluation of lameness in dogs		
Criterion	Degree	Clinical evaluation
Lameness	0	Normal ambulation
	I	Slight lameness
	II	Moderate lameness
	III	Severe lameness
	IV	Reluctance to get up, and does not walk >5 steps

McCarthy et al. (2007)

### Statistical analyses

The data were evaluated using descriptive statistics (means), with the results described as percentages of bone consolidation scores and lameness. Complications were reported as percentages and were related to clinical changes observed throughout the patient evaluations.

## RESULTS

Seventeen dogs underwent correction of MPL using the mTTT technique. All animals were classified with grade II MPL and had an estimated external tibial torsion of <20°. Three dogs were excluded because they were not brought in for the post‐operative radiographic follow‐up. Thus, 14 dogs met the inclusion criteria, and 15 stifles were operated. In the first consultation, all dogs had a history of intermittent lameness that was classified as grade I according to the scale adapted from McCarthy et al. (2007) (Table 2) and felt pain or discomfort when manipulating the stifle.

The case series comprised six male and eight female dogs with an average age of 25.5 ± 17.71 months and an average weight of 4.39 ± 2.87 kg. Six breeds were represented: Biewer (1/14), Boston terrier (1/14), Cavalier (1/14), Chihuahua (1/14), mixed breed (2/14) and German spitz (9/14). Thirteen dogs had unilateral grade II MPL and one bilateral; in five cases the right limb was affected and in ten cases the left. All dogs had an estimated external tibial torsion of <20°, with three cases at 5°, seven at 10° and five at 15°.

The realignment of the QEM and the stabilization of the patella in the trochlear groove were visually assessed and confirmed through stifle flexion/extension movements and external/internal rotation of the tibia relative to the femur at the conclusion of the technique in all patients (Fig 4). Steinmann pins with diameters of 2 and 2.5 mm were used to stabilize translations ranging from 2 to 3 mm. In one patient, a fracture of the osteotomized fragment occurred during the translation procedure, necessitating the application of a Kirschner wire through the TT to maintain stabilization (Fig 4). A patient underwent bilateral surgical treatment, with a 30‐day interval between the procedures. Bilateral desmotomy was performed in all patients, releasing the patella from the tension of the medial and lateral retinaculum. Wedge trochleoplasty was performed in four stifles.

**FIG 5:**
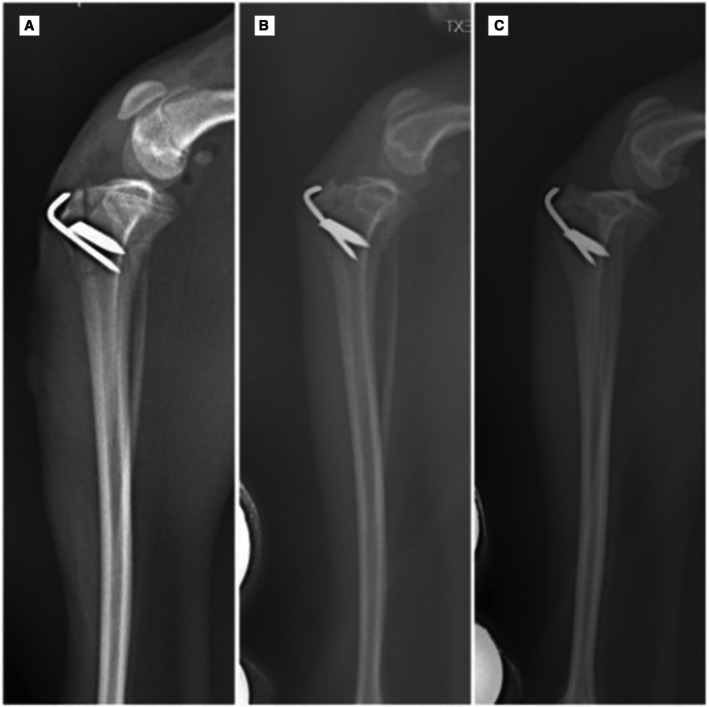
Radiographic follow‐up of a patient with tibial tuberosity fracture during surgery, necessitating the application of a Kirschner wire through the tibial tuberosity to maintain stabilization. (A) Left mediolateral radiograph from the immediate postoperative period showing a fracture of the base of the tibial tuberosity; (B) left mediolateral radiograph 30 days postoperatively showing good union; (C) Left mediolateral radiograph 60 days postoperatively showing excellent union.

In the immediate postoperative radiographic evaluation, it was confirmed that no implant had penetrated the caudal cortex of the tibia, and only one pin had been inserted below the insertion of the patellar ligament, with the remaining pins all inserted at the ideal location at the TT. In 14 stifles, a partial osteotomy line of the TT was observed, with no evidence of a fissure in the distal portion. One stifle exhibited a complete fracture of the base of the osteotomized fragment (Fig 5).

At the first evaluation, no patient presented pain, discomfort or crepitation during the evaluation of the operated stifle, and lameness was not recorded at the first evaluation. All patients had a stable patella, with no evidence of reluxation during the orthopaedic evaluation. No notable changes were identified in the surgical wounds, such as skin irritation, seroma or dehiscence. At 30 days post‐operative evaluation, 13 dogs presented lameness grade 0, with full support of the pelvic limbs. In one patient (7.14%), lameness grade I and slight discomfort were observed during the evaluation of the stifle with extension/flexion movements and joint palpation. All patients had a stable patella.

**FIG 6:**
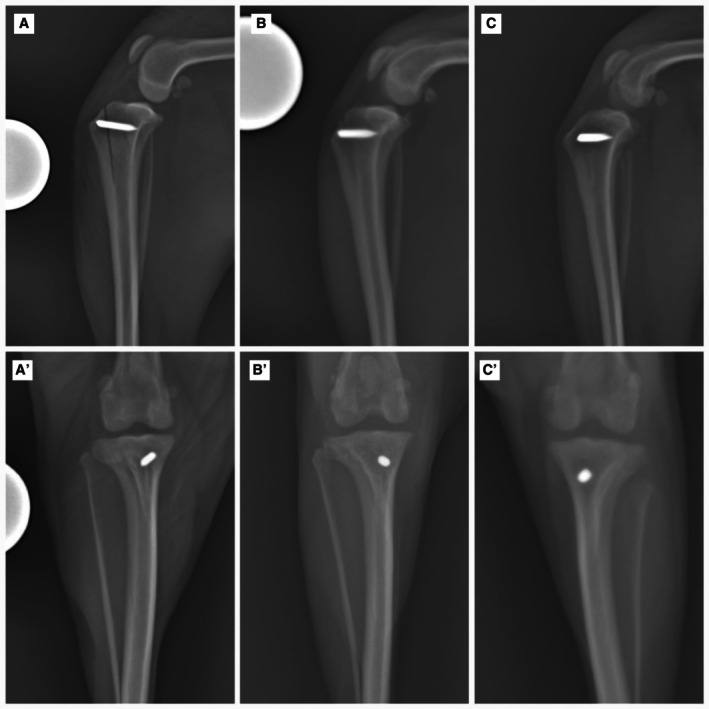
(A, A′) Mediolateral and craniocaudal radiograph of the immediate postoperative period of tibial tuberosity transposition with device (mTTT); (B, B′) mediolateral and craniocaudal radiograph 30 days postoperatively showing complete consolidation; (C, C′) mediolateral and craniocaudal radiograph 60 days postoperatively showing complete consolidation.

In the radiographic images at 30 days post‐operatively, complete bone consolidation was evident in 11 of 15 operated stifles (73.33%) (Fig 6B,B′); in one (6.66%), the consolidation was partial, with the osteotomy line evident only in the proximal portion; these 12 (80%) cases were classified as having excellent union; in one (6.66%), the osteotomy line was >50% and <75%, showing good union, and in two (13.33%), almost the entire osteotomy line was observed, indicating reasonable union. In one patient, implant migration was observed, but complete consolidation of the tuberosity was achieved, and thus a revision surgery was performed to remove the implant.

At 60 days, six patients returned for reevaluation, of which three had excellent union, one had good union, and two had reasonable union at 30 days. All dogs exhibited adequate weight‐bearing on the pelvic limbs (lameness grade 0), and none showed pain, discomfort, or crepitus upon stifle examination. Patellar luxation was not identified in any patient during the orthopaedic exam. Radiographic images of these patients demonstrated complete consolidation of the osteotomy (excellent union), with no implant migration observed in any patient (Fig 6C,C′).

**FIG 7:**
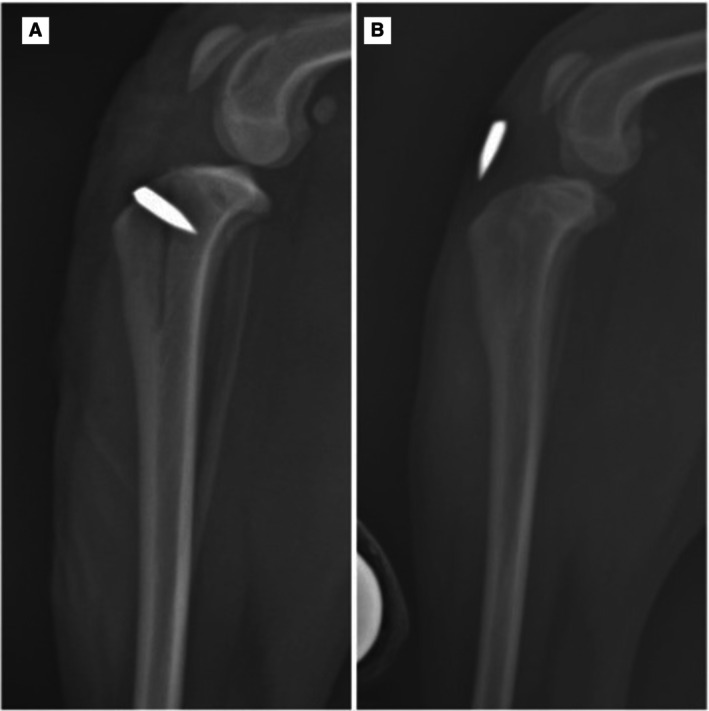
Radiographic follow‐up of a patient with implant migration. (A) Left mediolateral radiograph from the immediate postoperative period; (B) left mediolateral radiograph 30 days after the operation showing implant migration and complete consolidation of the osteotomy.

Two (13.33%) cases presented complications, one minor during surgery (6.66%) and one major post‐operative (6.66%), according to the classification proposed by Cook et al. (2010). In one dog, a fracture of the base of the fragment occurred during the TT translation procedure (Fig 5), which was promptly addressed with the application of a 1.2‐mm Kirschner wire through the TT; this complication had no clinical impact on the patient. In the other dog, implant migration was identified on the 30‐day radiographic evaluation (Fig 7), which was correlated with the lameness observed during the orthopaedic examination.

## DISCUSSION

The results of this study demonstrate that the mTTT technique is effective in treating grade II MPL, with excellent clinical results and a low complication rate, which preliminarily validates the initial hypothesis.

The results achieved may be due to the characteristics of the mTTT technique, which allows slow and controlled translation and is associated with a partial osteotomy that results in a more robust tibial crest and a more resistant distal bone pedicle. This procedure enables safer TTT, with greater/better healing potential (Filliquist et al., 2019; Leite, 2019; Petazzoni, 2015). From the mechanical point of view, the partial integrity of the tibial crest potentially increases the stability of the osteotomy and reduces the risk of fissures or avulsion of the osteotomized fragment, (Filliquist et al., 2019) unlike other techniques that apply some type of implant on the crest and perform total or near total osteotomy of the TT (Piermattei et al., 2006; Robins, 1990; Roush, 1993). According to Filliquist et al. (2019), the larger and wider base of the osteotomy performed in mTTT provides greater resistance to the tensile forces of the quadriceps muscle during locomotion. Given the non‐observation of fissure/fracture of the TT, it is believed that the mTTT technique benefited from such characteristics and prevented the occurrence of these complications reported in previous studies (Arthurs & Langley‐Hobbs, 2006; Bosio et al., 2017; Cashmore et al., 2014; Filliquist et al., 2019; Gallegos et al., 2016; Gibbons et al., 2006; Linney et al., 2011; Stanke et al., 2014).

Despite the more robust bone pedicle observed in mTTT, the acute translation of a more resistant tuberosity could potentially cause fissure/fracture in the crest, requiring the use of Kirschner wires or a tension band for TT fixation (Filliquist et al., 2019; Leite, 2019; Petazzoni, 2015). However, the low fracture rate of TT in the trans‐operative observed in this study, 6.66%, is most likely due to the characteristics of the device used to perform the technique, being comparable to other reported rates of 0.7% to 4% (Bosio et al., 2017; Cashmore et al., 2014; Gallegos et al., 2016). The device considers the elastic properties of bone tissue, which suffers deformity caused by a certain intensity of compression/traction without actually fracturing (Bala et al., 2011). The mechanism for mTTT allows slow and controlled translation of the TT through a certain tension applied on its medial/lateral surface, enabling the bone to adapt to the tension exerted on it and achieving the necessary displacement without the occurrence of fracture. The elastic property of bone tissue has also been considered in other techniques, such as the modified Maquet technique described by Etchepareborde et al. (2011).

Owing to the elastic capacity of the bone tissue at its base/pedicle, TTT requires the use of an implant strong enough to resist the forces of return to its original position, although the need for transfixation of the tuberosity itself is not observed, as seen in previously described TT transposition techniques (Cortina et al., 2023; Gibbons et al., 2006; Harasen, 2006; Kowaleski et al., 2017; Piermattei et al., 2006; Roush, 1993; Singleton, 1969; Stanke et al., 2014). Keeping the bone pedicle intact dispenses with the need for the anti‐rotational effect of multiple Steinmann pins / Kirschner wires as well as the compressive effect of tension bands (Filliquist et al., 2019; Leite, 2019; Petazzoni, 2015). Thus, the mTTT involves the use of a mechanical translation and temporary containment system followed by the application of a definitive implant, which is positioned adjacent to and below the frontal plane of the osteotomized TT, acting as a barrier to prevent it from returning to its original position (Leite, 2019; Petazzoni, 2015).

It is likely that the way in which the containment pin was positioned in the proximal tibia reduced the chances of skin irritation/inflammation and migration of the implant in the post‐operative period as it does not come into close contact with the skin and does not need to resist the tensile forces of the quadriceps muscle (Cortina et al., 2023; Filliquist et al., 2019; Petazzoni, 2015). Such complications have been highlighted in other studies that use conventional techniques most frequently and account for 7.7% to 24.6% of the cases (Cashmore et al., 2014; Cortina et al., 2023; Gallegos et al., 2016; Linney et al., 2011; Stanke et al., 2014). Stanke et al. (2014) recorded 24.6% implant migration, 13.8% implant failure and 18.2% skin irritation. In this case series, only one (6.66%) case of implant migration was observed, requiring its removal in the post‐operative period owing to discomfort and lameness after confirming bone consolidation of the transposition. We believe that this complication might have occurred owing to the use of a relatively short pin, which was not subcortical and adequately supported on the endosteum of the transcortical surface, leading to its migration during the post‐operative period.

Interestingly, no patellar reluxations were observed in the post‐operative period; these can occur in 8% to 48% of the cases in conventional surgeries and are one of the main reasons for surgical revision (Arthurs & Langley‐Hobbs, 2006; Cashmore et al., 2014; Gibbons et al., 2006; Linney et al., 2011). Although it is a more frequent complication in cases of grade III or IV PL, it can also occur in low‐grade corrections (Arthurs & Langley‐Hobbs, 2006; Cashmore et al., 2014; Perry & Déjardin, 2021). The characteristics of the technique, which have been discussed previously, favoured the good results in our case series, such as low rates of implant failure and skin irritation, rapid bone consolidation and early limb support without lameness. As recommended by Petazzoni (2015), our study was limited to patients with grade II PL, as higher external tibial torsion >20° may render the TTT inadequate. In such cases, a minimum of 50% contact between the osteotomy lines is not achieved, and alternative osteotomy techniques are recommended (Carrera et al., 2023; Silva et al., 2024). Thus, the sample characteristics might have influenced this finding, as the patients with lower degrees of PL tend to exhibit mild to moderate angular deviations of the tibia and femur, which, according to some authors, is correlated with a lower frequency of complications (Arthurs & Langley‐Hobbs, 2006; Cashmore et al., 2014; Perry & Déjardin, 2021). Additionally, the patients in our case series were mostly small, which is also associated with lower complication rates (Bosio et al., 2017; Gibbons et al., 2006; Stanke et al., 2014), although Arthurs and Langley‐Hobbs (2006) has stated that there is not enough evidence to corroborate such statements.

Although most previous studies involving TTT have not evaluated the consolidation time of osteotomy, it is believed that the patients in the present series presented early consolidation: 73.33% at 30 days and 100% at 60 days. When compared with other studies with techniques involving osteotomy of the proximal tibia, the results of bone consolidation appear to be superior, although these are distinct techniques and are not fully comparable. The means of bone consolidation in techniques such as tibial plateau levelling osteotomy (TPLO), modified TPLO, TPLO + TTT and lateral distal femoral closing wedge osteotomy + TTTT are between 30 and 105 days (Curuci et al., 2023; Flesher et al., 2019; Leonard et al., 2006). In techniques that are more similar to mTTT, such as TPLO and the modified TPLO, complete bone consolidation was seen at close to the 10th week in most cases (range: 6 to 15 weeks) (Flesher et al., 2019; Leonard et al., 2006). According to Etchepareborde et al. (2011), incomplete osteotomy of TT favours the healing process as it causes less damage to the periosteum on the tibial crest and preserves the blood supply, accelerating the consolidation of the osteotomy. Thus, we believe that the extensive preservation of the TT after osteotomy in mTTT favoured the rapid consolidation observed in this study.

Only a small number of patients were evaluated in our case series and were followed up for a relatively short period, which might have masked late complications such as implant migration and reluxation. Although the technique has been described since 2015 (Petazzoni, 2015), it has been little studied/evaluated, despite being considered a promising procedure for treating PL in patients with mild to moderate tibial torsion (Leite, 2019). The technique is recommended for patients with low‐grade PL (I or II), as in high‐grade cases with tibial torsion exceeding 20°, the required TT translation may render the technique impractical (Petazzoni, 2015). In such cases, other techniques are recommended to address more prominent bone deformities.

Although previous studies consider a variety of dogs in terms of age, weight, breed, and PL grade, which may interfere with the results and complication rates (Bosio et al., 2017; Cashmore et al., 2014; Cortina et al., 2023; Gallegos et al., 2016; Linney et al., 2011; Stanke et al., 2014), thus compromising a proper comparison with our cases, which only involved grade II PL, we believe that the results presented here, such as early bone consolidation and the absence of skin irritation, are potentially better. Additional studies should be conducted to evaluate its applicability in multimodal approaches in patients with higher degrees of PL.

The TTT technique modified with a slow and controlled translation device (mTTT) proved to be feasible and effective in realigning the extensor mechanism of the quadriceps and in maintaining patellar stability in patients treated in this study who exhibited grade II PL, tibial torsion of <20° and absent or minimal femoral varus. The patients showed rapid recovery, with a low rate of complications. No cases of reluxation were reported.

## Author contributions

**R. C. de Souza Faustino:** Conceptualization (equal); data curation (equal); formal analysis (equal); investigation (equal); methodology (equal); project administration (equal); visualization (equal); writing – original draft (equal). **E. H. P. Curuci:** Conceptualization (equal); data curation (equal); methodology (equal); project administration (equal); resources (equal); writing – review and editing (equal). **L. V. Costa:** Conceptualization (equal); formal analysis (equal); investigation (equal); methodology (equal); resources (equal); visualization (equal); writing – review and editing (equal). **B. W. Minto:** Conceptualization (equal); investigation (equal); methodology (equal); supervision (equal); validation (equal); writing – review and editing (equal). **L. G. G. G. Dias:** Conceptualization (equal); investigation (equal); supervision (equal); validation (equal); writing – review and editing (equal).

## Funding information

This research did not receive any specific grant from funding agencies in the public, commercial or not‐for‐profit sectors.

## Conflict of interest

No conflicts of interest have been declared.

## Data Availability

The data that support the findings of this study are available from the corresponding author upon reasonable request.
